# Supplementary material for: Iterative improvement in the automatic modular design of robot swarms
Source: PeerJ Comput Sci. 2020 Dec 7;6:e322. doi: 10.7717/peerj-cs.322 (PMC7924708; doi:10.7717/peerj-cs.322)
Supplement: Supplemental Information 3 [file peerj-cs-06-322-s003.zip › argos3/doc/api/standalone/a00538_source.html]

ARGoS: plugins/robots/generic/simulator/quadrotor\_position\_default\_actuator.cpp Source File


- Main Page
- Related Pages
- Namespaces
- Classes
- Files

- File List
- File Members

# plugins/robots/generic/simulator/quadrotor\_position\_default\_actuator.cpp

Go to the documentation of this file.

```
00001 
00007 #include "quadrotor_position_default_actuator.h"
00008 #include <argos3/core/utility/logging/argos_log.h>
00009 
00010 namespace argos {
00011 
00012    /****************************************/
00013    /****************************************/
00014 
00015    CQuadRotorPositionDefaultActuator::CQuadRotorPositionDefaultActuator() :
00016       m_pcQuadRotorEntity(NULL) {
00017    }
00018 
00019    /****************************************/
00020    /****************************************/
00021 
00022    void CQuadRotorPositionDefaultActuator::SetRobot(CComposableEntity& c_entity) {
00023       try {
00024          /* Get the quadrotor component */
00025          m_pcQuadRotorEntity = &(c_entity.GetComponent<CQuadRotorEntity>("quadrotor"));
00026          /* Check whether the control methods is unset - only one is allowed */
00027          if(m_pcQuadRotorEntity->GetControlMethod() == CQuadRotorEntity::NO_CONTROL) {
00028             /* Get the robot body */
00029             m_pcEmbodiedEntity = &(c_entity.GetComponent<CEmbodiedEntity>("body"));
00030             /* Set the position control method */
00031             m_pcQuadRotorEntity->SetControlMethod(CQuadRotorEntity::POSITION_CONTROL);
00032          }
00033          else {
00034             THROW_ARGOSEXCEPTION("Can't associate a quadrotor position actuator to entity \"" << c_entity.GetId() << "\" because it conflicts with a previously associated quadrotor actuator.");
00035          }
00036       }
00037       catch(CARGoSException& ex) {
00038          THROW_ARGOSEXCEPTION_NESTED("Error setting quadrotor position actuator to entity \"" << c_entity.GetId() << "\"", ex);
00039       }
00040    }
00041 
00042    /****************************************/
00043    /****************************************/
00044 
00045    void CQuadRotorPositionDefaultActuator::Init(TConfigurationNode& t_tree) {
00046       try {
00047          CCI_QuadRotorPositionActuator::Init(t_tree);
00048          Reset();
00049       }
00050       catch(CARGoSException& ex) {
00051          THROW_ARGOSEXCEPTION_NESTED("Initialization error in quadrotor position actuator.", ex);
00052       }
00053    }
00054 
00055    /****************************************/
00056    /****************************************/
00057 
00058    void CQuadRotorPositionDefaultActuator::SetAbsolutePosition(const CVector3& c_pos) {
00059       m_sDesiredPosData.Position = c_pos;
00060    }
00061 
00062    /****************************************/
00063    /****************************************/
00064 
00065    void CQuadRotorPositionDefaultActuator::SetRelativePosition(const CVector3& c_pos) {
00066       m_sDesiredPosData.Position = c_pos;
00067       m_sDesiredPosData.Position.Rotate(m_pcEmbodiedEntity->GetOriginAnchor().Orientation);
00068       m_sDesiredPosData.Position += m_pcEmbodiedEntity->GetOriginAnchor().Position;
00069    }
00070 
00071    /****************************************/
00072    /****************************************/
00073 
00074    void CQuadRotorPositionDefaultActuator::SetAbsoluteYaw(const CRadians& c_yaw) {
00075       m_sDesiredPosData.Yaw = c_yaw;
00076    }
00077 
00078    /****************************************/
00079    /****************************************/
00080 
00081    void CQuadRotorPositionDefaultActuator::SetRelativeYaw(const CRadians& c_yaw) {
00082       CRadians cYAngle, cXAngle;
00083       m_pcEmbodiedEntity->GetOriginAnchor().Orientation.ToEulerAngles(m_sDesiredPosData.Yaw, cYAngle, cXAngle);
00084       m_sDesiredPosData.Yaw += c_yaw;
00085    }
00086 
00087    /****************************************/
00088    /****************************************/
00089 
00090    void CQuadRotorPositionDefaultActuator::Update() {
00091       m_pcQuadRotorEntity->SetPositionControlData(m_sDesiredPosData);
00092    }
00093 
00094    /****************************************/
00095    /****************************************/
00096 
00097    void CQuadRotorPositionDefaultActuator::Reset() {
00098       m_sDesiredPosData.Position = m_pcEmbodiedEntity->GetOriginAnchor().Position;
00099       CRadians cYAngle, cXAngle;
00100       m_pcEmbodiedEntity->GetOriginAnchor().Orientation.ToEulerAngles(m_sDesiredPosData.Yaw, cYAngle, cXAngle);
00101       Update();
00102    }
00103 
00104    /****************************************/
00105    /****************************************/
00106 
00107 }
00108 
00109 REGISTER_ACTUATOR(CQuadRotorPositionDefaultActuator,
00110                   "quadrotor_position", "default",
00111                   "Carlo Pinciroli [ilpincy@gmail.com]",
00112                   "1.0",
00113                   "The quadrotor position actuator.",
00114                   "This actuator controls the position of a quadrotor robot. For a\n"
00115                   "complete description of its usage, refer to the\n"
00116                   "ci_quadrotor_position_actuator.h file.\n\n"
00117                   "REQUIRED XML CONFIGURATION\n\n"
00118                   "  <controllers>\n"
00119                   "    ...\n"
00120                   "    <my_controller ...>\n"
00121                   "      ...\n"
00122                   "      <actuators>\n"
00123                   "        ...\n"
00124                   "        <quadrotor_position implementation=\"default\" />\n"
00125                   "        ...\n"
00126                   "      </actuators>\n"
00127                   "      ...\n"
00128                   "    </my_controller>\n"
00129                   "    ...\n"
00130                   "  </controllers>\n\n"
00131                   "OPTIONAL XML CONFIGURATION\n\n"
00132                   "None for the time being.\n\n"
00133                   ,
00134                   "Usable"
00135    );
```

---

Generated on 10 Jul 2018 for ARGoS by 
 1.6.1
